# Impact of COVID-19 pandemic on viral keratitis

**DOI:** 10.6026/973206300222253

**Published:** 2026-04-30

**Authors:** Ankita Baghel, Aditi Mishra, Sanskriti Ukey, Pankaj Choudhary, Sachin Parmar

**Affiliations:** 1 Department of Ophthalmology, NandKumar Singh Chauhan Medical College, Khandwa, Madhya Pradesh, India; 2 Department of Ophthalmology, Shyam Shah Medical College, Rewa, Madhya Pradesh, India; 3 Department of Community Medicine, V.K.S. Government Medical College, Neemuch, Madhya Pradesh, India

**Keywords:** Keratitis, herpetic, COVID-19, vaccination, reactivation

## Abstract

COVID-19 has altered the epidemiology and clinical course of viral keratitis, with increasing evidence of herpesvirus reactivation after infection or vaccination. HSV keratitis remains most common in younger-to-middle-aged men, while HZO predominates in older adults and around one-fourth of HZO patients report current or prior COVID-19. HSV cases frequently recur and complications such as corneal opacity and neuropathic pain can cause vision outcomes ranging from normal to severe loss. Large recent studies suggest vaccination is associated with higher risk of new-onset and relapsing herpesviral keratitis, supporting antiviral prophylaxis for high-risk patients. Thus, screening around COVID-19 illness or vaccination and early, structured antiviral treatment reduces long-term visual impairment.

## Background:

COVID-19 has affected ocular health, including viral keratitis patterns and prevalence. Studies show that the pandemic affected infection incidence and severity [1]. Herpetic keratitis recurred shortly after mRNA COVID-19 vaccination, suggesting immune modulation and viral reactivation [2, 3]. Cytokine storm and transient immunosuppression caused by COVID-19 infection may reactivate herpes virus, increasing herpetic keratitis during the pandemic [4]. Additionally, the ocular surface expresses ACE2 receptors, which are SARS-CoV-2 entry points, suggesting a direct ocular infection route that may cause COVID-19-related keratitis or keratoconjunctivitis [5]. Keratitis can precede systemic symptoms in COVID-19 infection, suggesting ocular tropism. Such presentations suggest early ocular involvement and require ophthalmology [6]. Due to lockdowns and travel restrictions, the pandemic also hinders infectious keratitis treatment. Tertiary eye care centers found that delays in presentation to healthcare facilities led to advanced keratitis with large ulcers, corneal perforations and anatomical and functional failures [7]. Lack of donors disrupted corneal transplantation services, worsening outcomes [8]. The pandemic also increased acute angle-closure glaucoma cases, possibly due to COVID-19-related physiological stress and altered healthcare- seeking behavior. Although unrelated to viral keratitis, this trend shows how pandemics affect ophthalmologic diseases [9]. These findings show that the COVID-19 pandemic increased herpesviral reactivation, altered clinical presentations, complicated management due to healthcare disruptions and raised awareness of COVID-19's ocular manifestations. Ocular complications during and after COVID-19 infection or vaccination should be monitored, diagnosed and treated to reduce long-term visual morbidity [10]. The most important discovery is that both COVID-19 infection and vaccination can cause immune dysregulation that exposes latent ocular herpesviruses, necessitating vigilant ophthalmology monitoring and acyclovir dosage increases in high-risk patients during and after exposure. Therefore, it is of interest to understand the epidemiological and clinical trends of viral Keratitis in the age of COVID-19 is important to detect the disease early and provide the best treatment in this new clinical environment.

## Materials and Methods:

The present study title-Impact of COVID-19 pandemic on pattern of viral keratitis was conducted in department of ophthalmology S.S Medical College and Gandhi memorial Hospital, Rewa (M.P) from September 2022 to February 2024. Study consists 225 patients of keratitis from OPD and those admitted in eye department. Cases of viral keratitis selected on the basis of history and clinical Pattern.

## Study design:

Prospective study

## Inclusion criteria:

All cases of keratitis attending OPD and IPD.

## Exclusion criteria:

Corneal pathology other than keratitis causing visual impairment.

## Case selection:

The patients of viral keratitis were selected on the basis of history and clinical findings suggestive of viral keratitis. Dendritic ulcer was defined as a branching, linear lesion with terminal bulbs and swollen epithelial borders while pseudo-dendritic do not have terminal bulbs. The geographic ulcer was defined as an ulcer with enlarged, scalloped or geographic epithelial margins that penetrated the basement membranestromal keratitis was defined as stromal infiltration, either immune-mediated or necrotizing, with oedema. Endothelitis was defined as corneal stromal oedema without stromal infiltrate, but with keratic precipitates. Eyes which had typical clinical features, such as superficial punctate keratitis, dendrites, dendro-geographic stromal keratitis, endothelitis and kerato-uveitis were included in this study. The cases with vesicles of skin of face, eyelids and tip of nose with characteristic unilateral involvement of face associated with keratitis were noted.

## History:

Detailed history regarding demographic characters including name, age and sex, date of examination, central registration number and postal address were noted. History of COVID-19 status and COVID-19 vaccination were also noted. History of chief complains like pain and foreign body sensation, redness, watering, complaints of intolerance to light, diminution of vision was recorded. The presenting complaints were recorded with special reference to onset of symptoms, progression, seasonal variation, aggravating and relieving factors. Past history of each case noted with special attention to the similar episodes in the past and number of attacks (recurrences) and severity noted. History of any chronic illness likes tuberculosis, diabetes mellitus, malignancy and syphilis was recorded. History in relation to triggering factor for reactivation including prolonged fever, stress, use of any traditional eye medication and immunosuppressive drugs, UV light exposure, intra-ocular surgery, ocular trauma, use steroids were recorded. Duration between 1st and 2nd attack was noted and history of previous treatment of viral keratitis recorded. Season at the time of presentation recorded.

## Examination of skin over face:

Skin over face was examined for vesicles on forehead, cheeks and tip of nose with characteristic unilateral involvement.

## Ocular examination:

[1] Visual acuity: visual acuity was recorded at the time of presentation and during subsequent follow ups.

[2] Lids: lid edema, any discharge, vesicular eruptions on surrounding skin, lagophthalmos, entropion, ectropion and trichiasis were noted.

[3] Lacrimal apparatus: This was examined for evidence of infection. Possibility of chronic dacryocystitis was excluded.

[4] Conjunctiva: was examined for conjuncntival and cilliary congestion, subconjunctivalhaemorrhage, follicles or nodules.

## Cornea:

Corneal examination was done with the help of slit lamp bio- microscopy. The anterior surface of cornea was examined by focal illumination with broad beam. Corneal section with narrow slit beam used to focus different layers of cornea to access the depth of the corneal lesion, level of vascularisation and condition of endothelial and epithelial surfaces. The corneal ulcer examination was done under following heads-Site of the ulcer, size of the ulcer, shape of the ulcer, ulcer margins and floor of the ulcer, depth of the ulcer, surrounding area of ulcer & density of infiltration, vascularisation and degree of stromal oedema.

## Corneal sensations:

This test was done with the wisp of cotton. The patient was asked to look slightly upwards and cotton wisp was touched on the cornea as near as the centre as possible and then four quadrants of cornea. A reflex closure of eyes indicates normal sensation. Contact with eye lashes was always avoided. This test was compared with normal eye.

## Fluorescein staining of cornea:

This test was done in all the cases and repeated from time to time to demarcate the extent of the ulcer. Sterile fluorescein strips were used to avoid bacterial contamination. The lesions take up fluorescein is well highlighted under cobalt blue illumination of slit lamp.

## Pattern of corneal ulcer:

Examined with the help of slit lamp findings, pattern observed were -superficial punctate keratitis, dendritic, pseudo-dendritic, geographical, stromal, kerato-uveitis.

## Anterior chamber:

Anterior chamber was examined for aqueous cells, aqueous flare, keratic precipitates and presence of hypopyon by slit lamp.

## Iris:

The iris was examined for its color, pattern and exudates over the surface

## Pupil:

Its size, shape, margins and reaction were noted.

## Intraocular tension:

Intraocular tension was recorded with non-contact tonometer.

## Investigations:

The following investigations were routinely done.

## Corneal scrap for culture:

Culture and sensitivity for bacteria was done before starting any treatment. If the patient was already on treatment it was stopped for 24 hours before taking the swabs. The material for culture was obtained from the cornea which was anesthetized with a drop of 0.5% proparacaine; both the active edges as well as depth of corneal ulcer were scraped. Care was taken to avoid contact with the conjunctiva and lids. The material was sent to the microbiology laboratory of Department of Microbiology of S.S. Medical College Rewa.

## Relevant blood investigations:

Blood examination was done for -Hemoglobin, Total and differential WBC count and ESR. Blood sugar was tested in every case to rule out diabetes.

## Treatment:

## For HSV keratitis:

Ointment- 3% Acyclovir was instilled 5 times a day for 2 weeks. Cycloplegics was instilled in the form of atropine eye ointment (1%) twice a day in all cases. All the cases were supplemented with topical carboxymethyl cellulose eye drops, oral diclofenac sodium and multivitamins. Topical antiglaucoma eye drop was prescribed in cases of raised intraocular pressure. Oral acetazolamide 250 mg tablet three times a day along with syrup potassium hydrochloride was prescribed if require.

## For recurrent HSV keratitis:

Oral tablets-Acyclovir 400 mg twice a day for a month was given for patients with more than 3 episodes of recurrence and immune- compromised patients. Supportive treatment with oral analgesics, multivitamins and antacids were given. Topically eye ointment 3% acyclovir was instilled for five times a day for 7 days. Topical carboxymethyl cellulose eye drops and cycloplegics (atropine eye ointment) was prescribed. Topical antiglaucoma eye drop was prescribed in cases of raised intraocular pressure. Oral acetazolamide 250 mg tablet three times a day along with syrup potassium hydrochloride was prescribed if require.

## HZO Keratitis treatment:

Oral tablet- acyclovir 800 mg. five times a day was given for 10 days. Ointment-3% Acyclovir was prescribed 5 times a day for 2 weeks and Ointment- 5% Acyclovir was for skin vesicles for 2 weeks.

[1] In all cases cycloplegics was instilled in the form of atropine eye ointment (1%) twice a day.

[2] Topical carboxymethyl cellulose eye drops and oral multivitamin tablets were prescribed.

[3] Topical antiglaucoma eye drop was prescribed in cases of raised intraocular pressure. Oral acetazolamide 250 mg tablet three times a day along with syrup potassium hydrochloride was prescribed if required.

## Adenoviral treatment:

Topical steroids with antibiotic combination were used in tapering doses for 7 days. Cyclopentolate eye drops 3 times in a day. Systemic therapy-All the cases were supplemented with topical carboxymethyl cellulose eye drops, oral diclofenac sodium and multivitamins.

## Follow up:

All patients were followed up at first, 2nd week and 12th week. On each follow up visual acuity was recorded in all cases. Signs and symptoms were recorded in every follow up.

## Signs:

Lid oedema, conjunctival and cilliary congestion were noted. Slit lamp examination of the ulcer was done routinelyto note the size, shape, extent, depth, vascularization and surrounding area of the ulcer.

## Symptoms:

Foreign body sensations, redness, pain, diminution of vision, photophobia, watering, foreign body sensations were noted. Complications like corneal opacity, neovascularization, kerato- uveitis, neurotrophic keratitis and recurrence of the ulcer were recorded.

## Statistical analysis:

The collected data was fed in computer in MS excel. The clinical profile percentage-based comparison was done with previous study (conducted in 2010-2012)

## Results and Discussion:

There were 36 males (59%) and 25 females (41%), where the males were predominant in the subtypes. The age group that was mostly impacted was 21-40 years (39.3%), which was mostly related to HSV keratitis and the gender exhibited HZO keratitis which was higher in individuals aged above 60 years (64.3%), showing an age-specific predisposition pattern. The patient population in rural areas was 67.2 percent of the cohort population because of the inadequate access to healthcare and increased exposure to the risk in rural populations. In total, 16 patients (26.2) were concurrently infected with COVID-19 and the rates of two-dose vaccination were rather satisfactory (62.32) and only 3.3% were unvaccinated (Table 1 - see PDF). The most frequent clinical manifestation among subtypes was a superficial punctate lesion (41%), which was followed by dendritic ulcers in HSV cases (37.8%) and pseudo-dendritic types in HZO cases (64.3) are pathognomonic features of the underlying viral etiology (Table 2 - see PDF). The geographical lesions and stromal lesions were less common. Mostly unilateral presentation was evident (93.4%) and bilateral involvement was only seen in four. In terms of complications of the disease, the most common was corneal opacity (19.7%), then neovascularization (22.9) and recurrence, which was also quite frequent in the case of HSV (62.2%), indicating the presence of a latent virus (Table 3 - see PDF). HZO cases exhibited unique neuro-ophthalmic complications such as trigeminal neuralgia (71.4%) and neurotrophic keratitis (35.7) demonstrating the unpredictable nerve involvement as is characteristic of herpes ophthalmicus. Visual acuity results were not consistent between subgroups at final follow-up (n = 55) (Table 4 - see PDF). There was normal vision (25.5% 6/6) and most patients (41.8) had mild-moderate visual limitation (6/9 - 6/18). A further 25.5% had a mid-range of acuity 6/24-6/60 and a minor group (7.2) with severe visual loss (<6/60) in the majority cases due to corneal scarring or secondary neuropathic alterations. All in all, the statistics show that HSV keratitis has been the most prevalent case of infectious keratopathy, mostly in younger and middle-aged patients, whereas HZO keratitis has been more widespread among older, systemically immunocompromised individuals. The proportion of cases of post-COVID-19 viral keratitis (26.2 percent) is relatively high, which could be associated with reactivation of the latent viral infections in the context of dysregulation of immunity. The findings from the current study align with existing literature that highlights HSV keratitis as the predominant infectious keratopathy in younger and middle-aged populations, usually in males, while HZO keratitis tends to affect older, immuno-compromised individuals predominantly in rural settings. The male predominance and most affected age group of 21-40 years for HSV keratitis are comparable to the large cohort study conducted at a tertiary center in India by Das, Anthony Vipin *et al.* [10] which reported that 65% of HSV keratitis patients were male with a median age of 40 years and more than half came from rural populations. The rural predominance observed in this study also reflects disparities in healthcare access noted in other Indian studies, emphasizing the need for targeted rural eye care services [11]. The high recurrence rate for HSV keratitis (62.2%) concurs with global data showing HSV's propensity for latency and reactivation, leading to recurrent keratitis episodes and cumulative corneal damage. HZO's noted neuro-ophthalmic complications such as trigeminal neuralgia and neurotrophic keratitis reflect the literature describing the severe nerve involvement characteristic of herpes zoster ophthalmicus, particularly in older adults [12, 13-14]. The observed 26.2% concurrence of viral keratitis with COVID-19 infection supports emerging evidence of increased herpesvirus reactivation linked to the immune dysregulation induced by COVID-19 infection and vaccination [1]. A nationwide cohort study demonstrated a significantly higher risk of herpesviral keratitis following COVID-19 vaccination, especially in males and older age groups, consistent with the findings here. Similarly, clinical case reports and series have documented reactivation of herpetic keratitis shortly after COVID-19 vaccination [2]. The association suggests that both systemic viral illness and immune-modulating interventions during the pandemic may act as triggers for viral keratitis reactivation [3]. Visual acuity outcomes reported in this study-with about 25.5% maintaining normal vision at final follow-up and a noticeable proportion suffering moderate to severe visual impairment-are consistent with previous epidemiological reports emphasizing the substantial morbidity burden due to viral keratitis. The visual impairment is often secondary to corneal scarring and neuropathic changes post-inflammation [13].

## Conclusion:

Viral keratitis trends changed during COVID-19, with more recurrences and complications. COVID-19 infection or vaccination may trigger herpetic keratitis reactivation, increasing HSV/HZO cases-HSV more in younger adults and HZO more in older, immuno compromised people. Thus, visual outcomes improve with early diagnosis, patient education and close follow-up after infection or vaccination.

## References

[1] Lee TE (2025). Cornea..

[2] Richardson-May J (2021). BMJ Case Reports..

[3] Tsatsos M (2025). Vision (Basel)..

[4] Maldonado MD (2021). Epidemiology and infection..

[5] Bao Q (2025). Scientific reports..

[6] Christy JS (2021). Cornea..

[7] Hoffman JJ (2022). Front Med (Lausanne)..

[8] Daher ND, Zeba AS (2023). BMC ophthalmology..

[9] Zhu R (2022). International journal of ophthalmology..

[10] Das AV (2023). International Ophthalmology..

[11] https://www.ncbi.nlm.nih.gov/books/NBK545278/.

[12] Das N (2022). Indian journal of ophthalmology..

[13] McCormick I (2022). Ophthalmic Epidemiology..

[14] Lin YH (2026). QJM..

